# Anterior bone tunnel position increases meniscus migration in medial meniscus posterior root repair: A cadaveric study of suture length changes

**DOI:** 10.1002/jeo2.70028

**Published:** 2024-09-30

**Authors:** Kazuya Nishino, Yusuke Hashimoto, Takuya Kinoshita, Ken Iida, Shuko Tsumoto, Hiroaki Nakamura

**Affiliations:** ^1^ Department of Orthopaedic Surgery Osaka Metropolitan University Graduate School of Medicine Osaka Japan; ^2^ Osaka University of Health and Sport Sciences Graduate School of Sport Sciences Osaka Japan; ^3^ Department of Orthopaedic Surgery Saiseikai Nakatsu hospital Osaka Japan

**Keywords:** bone tunnel, cadaver, medial meniscus posterior root tear, meniscus

## Abstract

**Purpose:**

This study investigated differences in the migration of meniscus sutured with pull‐out sutures for treating medial meniscus posterior root tears (MMPRTs) according to the bone tunnel position, using cadaveric knees.

**Methods:**

Six knees of three donors fixed using Thiel's method were included in this study. The MMPRTs were created, and a single suture was performed at the torn meniscus using an arthroscopic procedure. The suture was pulled out through the tibial bone tunnel, and the meniscus displacement was measured as the change in length during 0–120° of knee flexion. Three types of bone tunnels (anatomical, anterior and posterior) were created for each knee, and the sutures were pulled out of each tunnel three times. After completing all measurements, the proximal tibia was extracted and micro‐computed tomography was performed to evaluate the tunnel position.

**Results:**

A significantly smaller change in suture length was observed in the posterior group compared to the other two groups (anatomical group, 5.17 ± 1.8 mm; anterior group, 7.50 ± 3.2 mm; posterior group, 1.17 ± 1.0 mm; *p* > 0.01). In addition, a significant correlation between the anteroposterior tunnel position and suture length change was observed (*r* = −0.720; *p* = 0.001).

**Conclusions:**

When pull‐out sutures were used to repair MMPRTs, the suture length change was approximately 5 mm during knee flexion and extension when the bone tunnel was located at the anatomical attachment site. This change was larger when the tunnel position was anterior, and smaller when the tunnel position was posterior.

**Level of Evidence:**

LEVEL Ⅲ case–control study

**Abbreviations**
CT: computed tomography
MMPRT: medial meniscus posterior root tear
MRI: magnetic resonance imaging

## BACKGROUND

A medial meniscus posterior root tear (MMPRT) is a radial tear in the posterior attachment of the medial meniscus. It occurs with degeneration of the meniscus and results in extrusion of the meniscus [2]. MMPRTs increase the contact pressure of the knee joint, and untreated MMPRTs increase the risk of knee osteoarthritis and idiopathic osteonecrosis [11, 16]. While several surgical treatment methods have been reported, arthroscopic surgery involving a bone tunnel created using pull‐out sutures is often used as a treatment option; however, although there have been some reports of stable clinical outcomes with pull‐out suture repair techniques [7], incomplete or nonhealing of the repaired area is often observed during second‐look surgery [10]. Although varus alignment and older age are risk factors for poor outcomes [3, 12], reducing the risk of suture cut‐out remains important because of the degenerative nature of the tear. Daney and colleagues recommended creating a bone tunnel in the anatomical position [4]. As the meniscus usually moves anteriorly and posteriorly as the knee flexes and extends [1], the amount of meniscus movement may increase depending on the position of the bone tunnel, causing excessive stress on the suture. However, few studies have examined the dynamic translation of the suture in relation to the tunnel location; therefore [8], a consensus has not been reached. This study aimed to investigate the differences in sutured meniscus migration according to the bone tunnel position created with pull‐out sutures for MMPRTs, using cadaveric knees. We hypothesized that posterior positioning of the bone tunnel reduces the risk of length change because suture translation and stress on the sutures are decreased.

## MATERIALS AND METHODS

**Figure 1:**
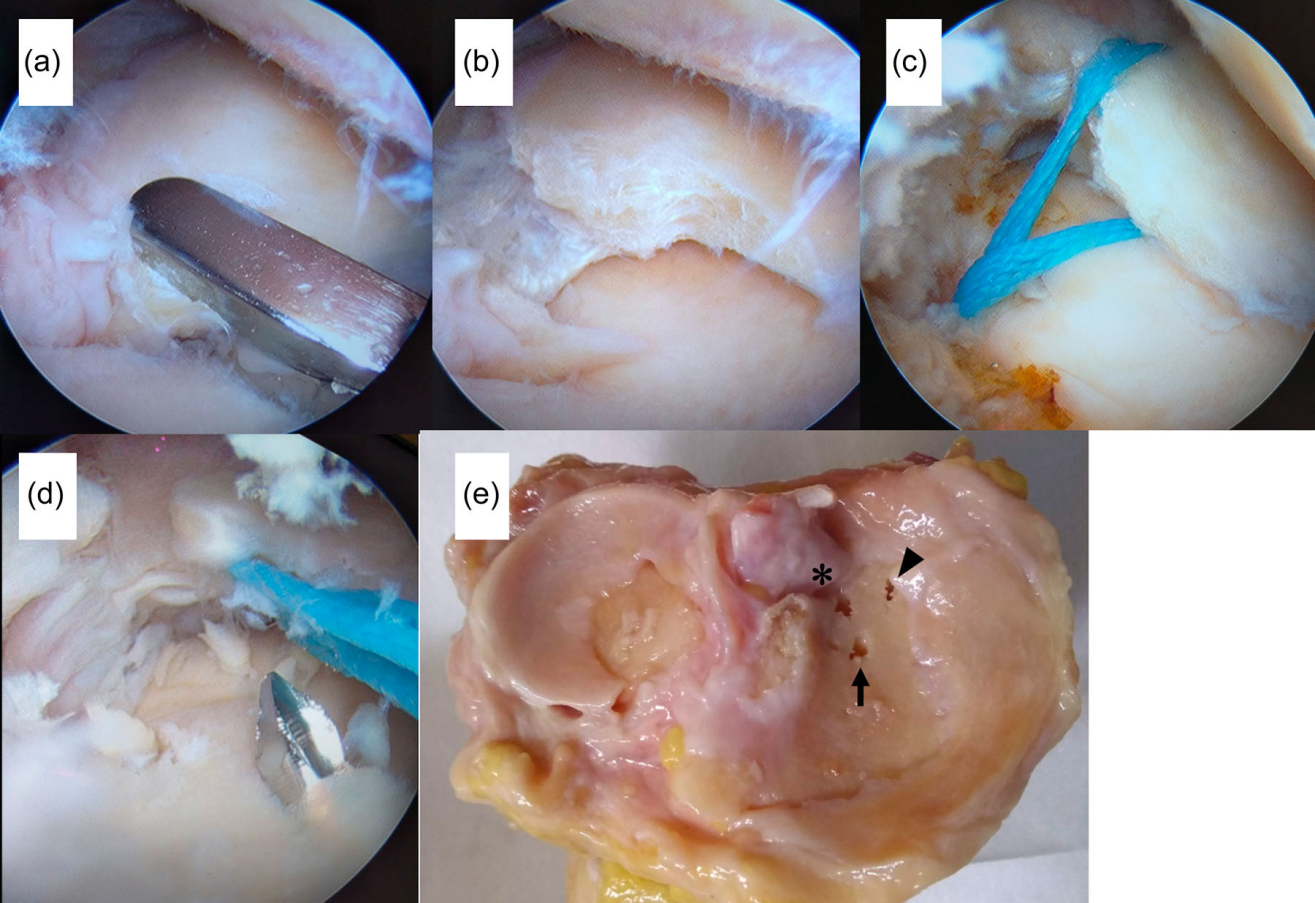
Arthroscopic and macroscopic photographs of medial meniscal posterior root repair. (a) The root tear was created using arthroscopic forceps. (b) Confirmation of the tear. (c) A single suture was pulled out through the bone tunnel. (d) After measurement, another tunnel was created anterior to the anatomical position. (e) Resected tibial plateau without medial meniscus showing anatomical (asterisk), anterior (arrow) and posterior (arrowhead) bone tunnels.

This study was performed in line with the principles of the Declaration of Helsinki. Approval was granted by the Ethics Committee (No. 4058). Six knees of three donors (84‐year‐old male, 92‐year‐old female and 62‐year‐old male) fixed using Thiel's method were included in this study. The Thiel's method preserves cadavers by soaking them in a unique solution of balsams, salts and formaldehyde, leading to long‐term storage with better flexibility and elasticity of tissues [14]. The subject was placed in the supine position, the limb was fixed with a leg holder and the examination was conducted using an arthroscopic system to simulate an actual surgical procedure. Anterolateral and anteromedial portals were created, and standard arthroscopic observation was performed. The knee pathologies of all samples are shown in Table S1. Briefly, all samples exhibited no ligament damage or meniscal tears, including MMPRT. Degeneration of the cartilage was observed. The posterior root of the medial meniscus was identified, and the MMPRT was created using a narrow biter 5 mm medial to the attachment site. When the joint space was narrow, the additional distal attachment of the medial collateral ligament was released. A single suture was initially placed at the posterior root using FIRSTPASS MINI (Smith & Nephew) and ULTRATAPE (Smith & Nephew) and was subsequently pulled out through a bone tunnel created using an MMPRT guide and 2.4‐mm K‐wire. The pull‐out through the bone tunnel was performed using the relay technique. Three types of tunnels were created in each knee: anatomical (anatomical attachment site); anterior (>5 mm medial and anterior to the anatomical position); and posterior (>5 mm medial and posterior to the anatomical position) (Figure 1). The exit sites for the bone tunnels on the anterior surface of the tibia were created medially. Specifically, the guide was placed about 2 cm medial from the tibial tuberosity and 1.5 cm superior to the pes anserinus. Using a parallel guide, three bone tunnels were made to ensure that there was no connection between the tunnels, whether on the joint surface or the anterior tibia.

**Table 1:** Tunnel position and total length change among three types of bone tunnel.

Tunnel position	Lateromedial position (% ± SD)	Anteroposterior position (% ± SD)	Total length change (mm ± SD)
Anatomic	36.9 ± 3.5	75.6 ± 2.4	5.2 ± 1.8
Posterior	27.2 ± 5.2	81.1 ± 4.3	1.2 ± 1.1
Anterior	33.2 ± 4.4	64.2 ± 4.0	7.5 ± 3.2
*p* Value	0.002	0.006	0.006

**Figure 2:**
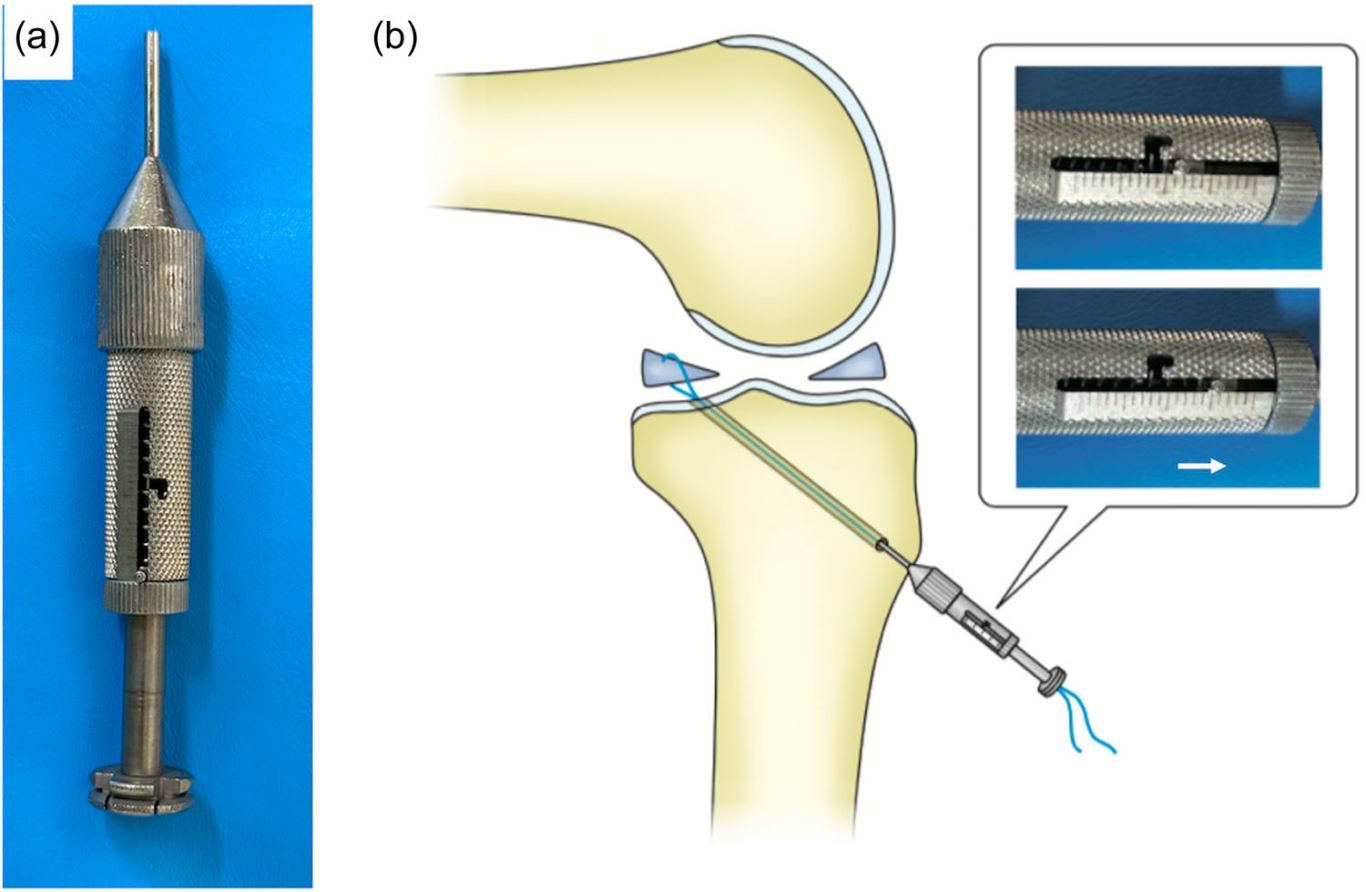
(a) Isometric positioner. (b) Measurement of the suture length change at 0–120° of knee flexion using an isometric positioner.

**Figure 3:**
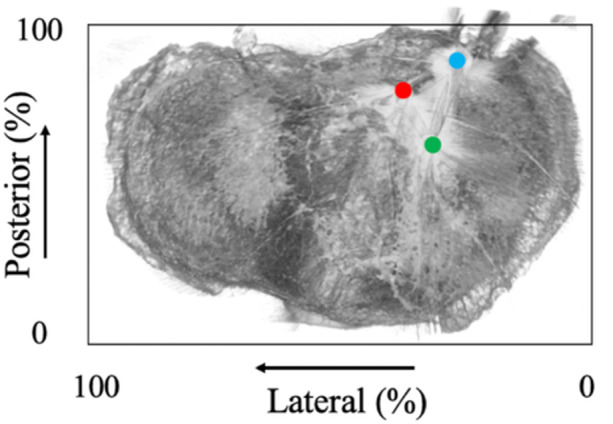
Evaluation of the bone tunnel position using three‐dimensional (3D) computed tomography (CT) of the tibial plateau: anatomical (red dot), anterior (green dot) and posterior (blue dot) bone tunnels are shown.

An isometric positioner (Smith & Nephew) was fixed to the suture from the distal tibial tunnel. The amount of change in suture length was measured at 0–120° of knee flexion, with 90° of knee flexion as the reference (±0 mm) (Figure 2). Then, the suture was pulled back inside the knee joint and pulled out again into another tunnel, and the same measurement was repeated. The procedure for knee flexion and extension involved placing the cadaver on a table, with the examiner adjusting the knee and concurrently noting the isometer's readings. The angles of flexion were also confirmed by an assistant employing a goniometer from a lateral perspective. After obtaining all measurements, the proximal tibia was dissected and computed tomography (CT) (LaTheta LCT‐100A scanner; Hitachi‐Aloka Medical Ltd.) was performed to evaluate the tunnel position (Figure 3). The intra‐articular position of the tunnel was quantified as lateromedial and anteroposterior percentages, based on the report by Fujii et al. [5] Briefly, the relative position from the anterior edge of the bone tunnels to the anteroposterior width of the tibial plateau was assessed as the anteroposterior position (%), and similarly, the relative position from the medial edge to the mediolateral width was assessed as the lateromedial position (%).

### Statistical analysis

The Kolmogorov–Smirnov test was conducted for the continuous variables, resulting in the rejection of normality. Therefore, all measurements of length changes and tunnel positions were compared using the Friedman test and the post hoc Wilcoxon test. The association between the total meniscal movement and the anteroposterior and lateromedial positions of the bone tunnels was analyzed using Spearman's correlation coefficient. A power analysis was performed with the power, *α*‐level, difference and SD set at 0.8, 0.01, 6.5 and 3.2, respectively, according to total length change. To detect a difference in length change between the posterior group and the anterior group with the Wilcoxon test, a minimum of seven samples was required. All hypotheses were tested assuming a significance level of 0.05 and a two‐sided alternative hypothesis. All analyses were performed using SPSS version 23 (IBM Corp.).

## RESULTS

**Figure 4:**
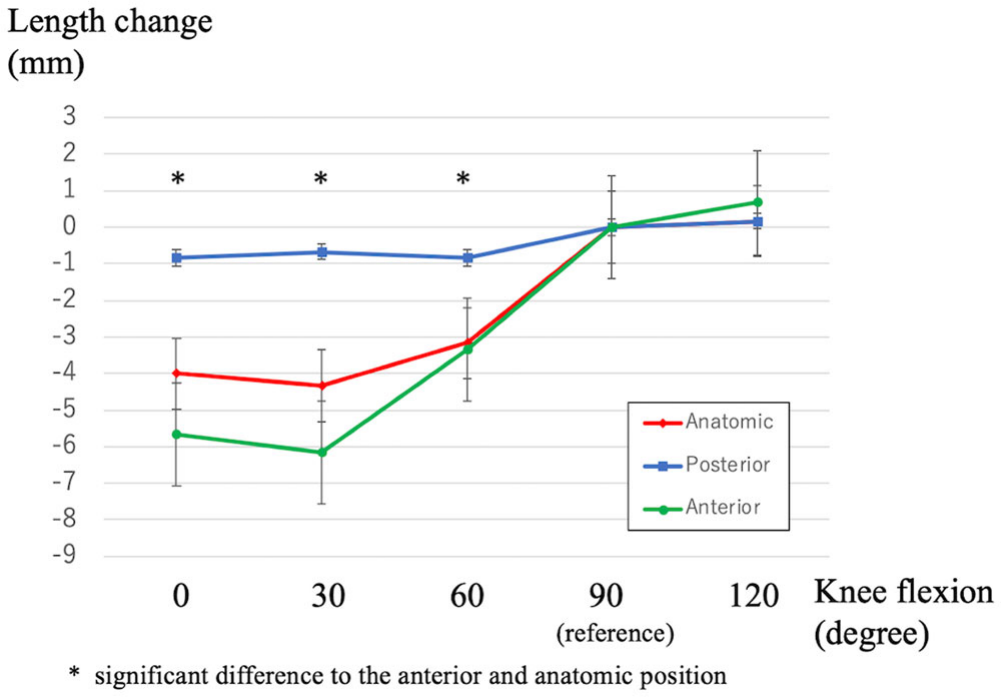
Suture length change with three types of bone tunnels.

**Figure 5:**
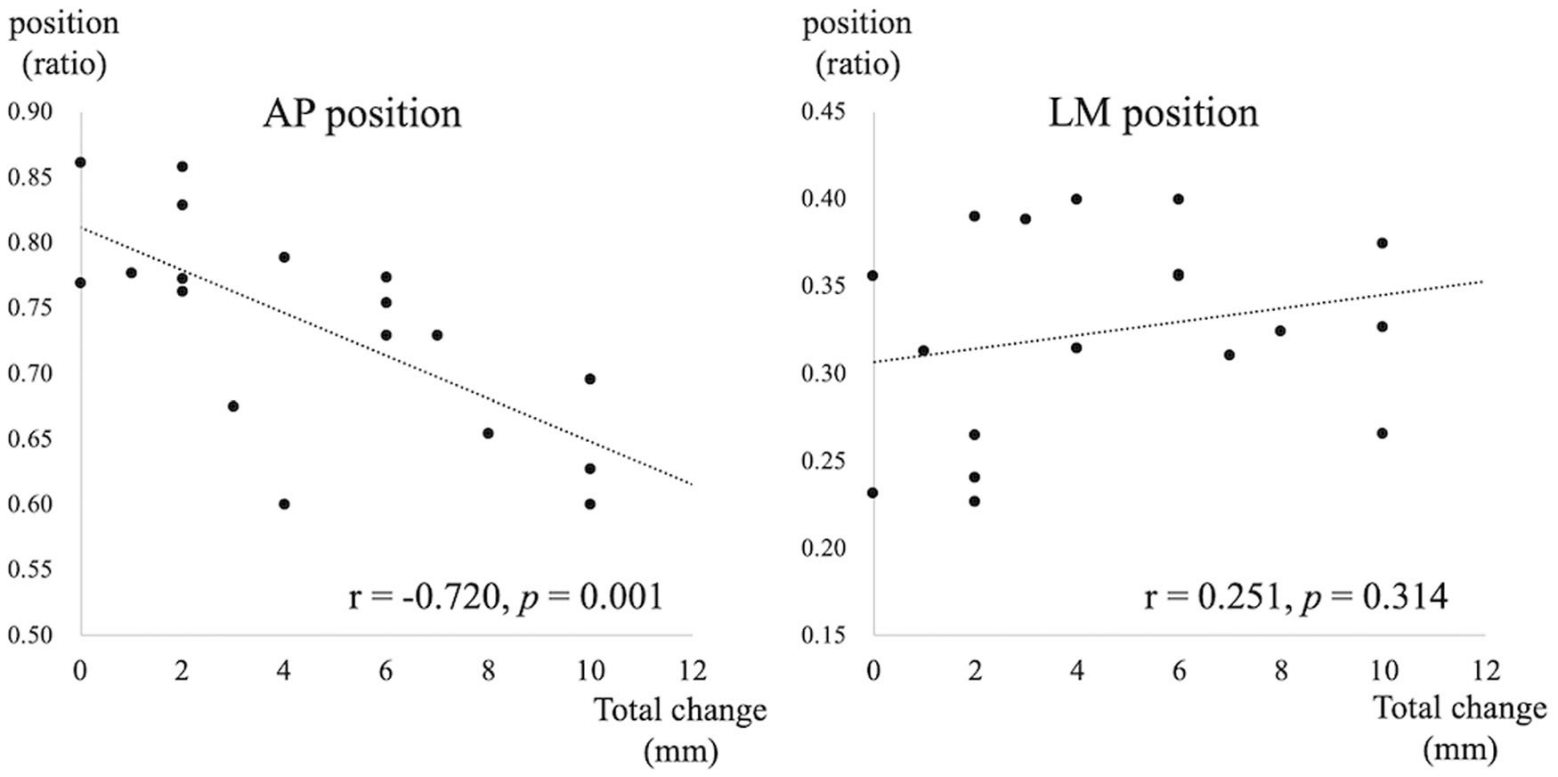
Relationship between the anteroposterior and lateromedial positions of the bone tunnel and suture length change.

The length change according to the knee flexion angle for each bone tunnel (Figure 4) was translated using knee flexion in the anatomical and anterior groups, suggesting increased loading on the suture site. The anterior group showed the largest length change of the meniscus (mean, 7.50 ± 3.2 mm), whereas the posterior group showed isometric movement (mean length change, 1.17 ± 1.0 mm). The anatomical group had a total length change of 5.17 ± 1.8 mm. The posterior group exhibited significantly less change in the total length than the other two groups (Table 1). The positions of the bone tunnels according to the lateromedial and anteroposterior percentages were as follows: anatomical (36.9 ± 3.5% and 75.6 ± 2.4%, respectively), posterior (27.2 ± 5.2% and 81.1 ± 4.3%, respectively), and anterior (33.2 ± 4.4% and 64.2 ± 4.0%, respectively). The average values, standard deviations, 95% confidence intervals and *p* values for all parameters are shown in Table S2. A significant correlation was observed between the anteroposterior tunnel position and length change (*r* = −0.720; *p* = 0.001). No significant difference between the lateromedial tunnel position and total length change was observed (*r* = −0.251; *p *= 0.314) (Figure 5).

## DISCUSSION

The most important findings of this study were as follows: when the bone tunnel was located at the anatomical attachment, the length change was approximately 5 mm; the length change was larger when the tunnel was located anteriorly, and smaller when the tunnel was located posteriorly. In this study, given that three different bone tunnels were created in the same cadaver knee and the length changes were compared, it was possible to determine that the factor that influenced the length change was the anteroposterior bone tunnel position when the kinematics of flexion and extension aligned. When similar clinical studies were conducted in the past, only one bone tunnel could be created for each knee; therefore, inevitably, the effects of individual differences may be reflected in the results of such studies. Regarding the anatomical location of the medial meniscal root, Furumatsu and colleagues reported that the tunnel position was 78.5% posterior and 39.4% lateral during a three‐dimensional‐CT study [6]. In the present study, the tunnel position in the anatomical group was 76% posterior and 36% lateral, thereby reproducing a tunnel position similar to that in previous studies.

Vedi and colleagues conducted a magnetic resonance imaging (MRI) study and reported that the amount of displacement of the medial meniscus posterior horn during normal knee flexion was approximately 4 mm, regardless of whether an axial load was applied [15]. Even a healthy, uninjured medial meniscus allows a certain amount of movement during knee flexion and extension. In contrast, Okazaki and colleagues found a displacement of 6.3 mm between 10° and 90° of knee flexion in patients with MMPRTs during 3D‐MRI [13].

Pulled sutures may continue to be subjected to dynamic stress postoperatively. Hiranaka and colleagues measured suture displacement during suture pull‐out in patients with MMPRT and found that it ranged from 3.9 to 4.8 mm during flexion and extension [8]. However, the amount of suture displacement based on the position of the bone tunnel was not investigated, and the influence of the tunnel position on the amount of displacement was not identified. Kamatsuki and colleagues reported that an anatomical tunnel position decreased the posterior meniscal displacement after MMPRT repair [9]. Most of the cases in that study, however, were in the antero‐medial position, and more posterior tunnel placement was not evaluated. In the present study, we found a significant correlation between the position of the bone tunnel in the anteroposterior direction and meniscal displacement. During actual surgical procedures, when it is difficult to position the bone tunnel in the posterior direction, resulting in the tunnel being created in the anterior direction, the stress on the suture after surgery increases, suggesting an increased risk of cut‐out. Isometric movement was observed when the tunnel was created posterior to the anatomical position, suggesting that creating a posterior bone tunnel results in less stress. Based on the results of this study, surgeons should consider the risk of suture cut‐out, and the change in length caused by the knee flexion angle should be evaluated before the pull‐out suture is fixed. In terms of reducing the stress on the root repair, we recommended placing the bone tunnel further posterior in comparison to the anatomical footprint.

This study had several limitations. First, the sample size was small. Although the alpha error was set stringently, the power analysis showed that the required sample size fell short by one case. Second, the cadavers were older than the age at which surgery for MMPRT is most likely to be indicated. Third, the kinematics of the knees in this study may have differed from normal knee kinematics because of the use of cadaveric knees. The Thiel method retains the flexibility and movement of joints, making it suitable for biomechanical testing; however, it can lead to uneven preservation compared to other methods. This nonuniformity may cause variations in test results, potentially affecting the reliability of the findings. Fourth, the study did not directly measure meniscal movement but rather assessed the length of the suture, which admits the potential for device‐related measurement errors. Fifth, the contact area and pressure were not evaluated. Although the creation of the posterior bone tunnel was associated with less dynamic stress on the suture, whether the knee joint protection function is reduced requires further investigation. Finally, the actual cut‐out was not evaluated. Creation of the anatomical bone tunnel resulted in a length change of 4.8 mm; therefore, whether cut‐out occurs during flexion and extension after fixation of the pulled‐out suture may be more directly evaluated. Nevertheless, this study clarified the relationship between the bone tunnel position and dynamic meniscal displacement. The optimal fixation angle remains unknown because this study did not directly assess the fixation angle and tension.

## CONCLUSION

The relationship between the position of the bone foramen and change in the suture length of pull‐out sutures for MMPRT was investigated using cadaveric knees. When the bone tunnel was located at the anatomical attachment site, the change in length was approximately 5 mm during knee flexion and extension. This change was larger when the tunnel position was anterior, and smaller when the tunnel position was posterior.

## AUTHOR CONTRIBUTIONS

*Conception and design*: Kazuya Nishino. *Drafting of the article*: Kazuya Nishino. *Conception, and critical revision of the article for important intellectual content*: Yusuke Hashimoto. *Interpretation of data*: Takuya Kinoshita. *Revising the manuscript and data collection*: Ken Iida. *Data collection*: Shuko Tsumoto. *Conception and design, final approval of the article*: Hiroaki Nakamura.

## CONFLICT OF INTEREST STATEMENT

The authors declare no conflict of interest.

## ETHICS STATEMENT

This study was performed in line with the principles of the Declaration of Helsinki. Approval was granted by the Ethics Committee (No. 4058).

## Supporting information

Supporting information.

## Data Availability

The data sets used and/or analyzed during the current study are available from the corresponding author on reasonable request.
