# Supplementary material for: Anterior bone tunnel position increases meniscus migration in medial meniscus posterior root repair: A cadaveric study of suture length changes
Source: J Exp Orthop. 2024 Sep 30;11(4):e70028. doi: 10.1002/jeo2.70028 (PMC11440485; doi:10.1002/jeo2.70028)
Supplement: Supplementary file 1 — Supporting information. [file JEO2-11-e70028-s001.docx]

| Supplemental Table 1. Knee pathologies of the samples | | | | | | | | |
| --- | --- | --- | --- | --- | --- | --- | --- | --- |
| sample | age | Gender | Contracture | ACL | MMPRT | Degeneration of  medial meniscus | ICRS for  Femoral cartilage | ICRS for  Tibial cartilage |
| No. 1 | 84 | Male | Non | Intact | Non | Intact | Ⅱ | Ⅱ |
| No. 2 | 84 | Male | Non | Intact | Non | Intact | Ⅱ | Ⅱ |
| No. 3 | 92 | Female | Non | Intact | Non | Fluttering | Ⅳ | Ⅳ |
| No. 4 | 92 | Female | Non | Intact | Non | Fluttering | Ⅳ | Ⅳ |
| No. 5 | 62 | Male | Non | Intact | Non | Fluttering | Ⅲ | Ⅲ |
| No. 6 | 62 | Male | Non | Intact | Non | Fluttering | Ⅲ | Ⅲ |
| ACL: Anterior Cruciate Ligament, MMPRT: Medial Meniscus Root Tear, ICRS:　International Cartilage Repair Society. | | | | | | | | |

| Supplemental Table 2. Length changes and tunnel positions | | | | | | | | | |
| --- | --- | --- | --- | --- | --- | --- | --- | --- | --- |
|  |  | 0 degree | 30 degree | 60 degree | 90 degree | 120 degree | total change | LM position | AP position |
| Tunnel position  ±SD, [95%CI] | Anatomical | -4.0±1.4  [-5.5, -2.5] | -4.3±1.6  [-6.0, -2.6] | -3.2±0.7  [-3.9, -2.5] | 0 | 0.2±0.4  [-0.2, 0.6] | 5.2±1.8  [3.3, 7.1] | 0.37±0.03  [0.34, 0.40] | 0.76±0.02  [0.74, 0.78] |
|  | Posterior | -0.8±1.0  [-1.8, 0.2] | -0.7±1.2  [-2.0, 0.6] | -0.8±1.0  [-1.8, 0.2] | 0 | 0.2±0.4  [-0.2, 0.6] | 1.2±1.0  [0.2, 2.2] | 0.27±0.05  [0.22, 0.32] | 0.81±0.04  [0.77, 0.85] |
|  | Anterior | -5.7±3.0  [-8.8, -2.6] | -6.2±2.6  [-8.9, -3.5] | -3.3±1.2  [-4.6, -2.0] | 0 | 0.7±1.5  [-0.9, 2.3] | 7.5±3.2  [4.1, 10.9] | 0.33±0.04  [0.29, 0.37] | 0.64±0.04  [0.60, 0.68] |
| P value | Freedman test | 0.006 | 0.006 | 0.016 | 1 | 0.949 | 0.006 | 0.002 | 0.006 |
|  | Anatomical vs Anterior | 0.030 | 0.043 | 0.021 | NA | NA | 0.043 | 0.001 | 0.248 |
|  | Anatomical vs Posterior | 0.386 | 0.248 | 0.885 | NA | NA | 0.248 | 0.083 | 0.043 |
|  | Anterior vs Posterior | 0.002 | 0.001 | 0.014 | NA | NA | 0.001 | 0.083 | 0.001 |
